# Grand challenges in oral health and nutrition: We are what we eat

**DOI:** 10.3389/froh.2022.999817

**Published:** 2022-08-24

**Authors:** Anwar T. Merchant

**Affiliations:** Department of Epidemiology and Biostatistics, University of South Carolina, Columbia, SC, United States

**Keywords:** oral health, nutrition, diet, health outcomes, socioeconomic disparities, food insecurity

## We are what we eat

Research linking diet and health outcomes, and oral and systemic health has proliferated in the last two decades. The notion that poor oral health leads to poor diet is intuitive, and is supported by studies showing that individuals with missing or decayed teeth or oral soft tissue lesions are more likely to replace foods that are hard to chew, such as fresh fruits and vegetables, with less healthy alternatives such as processed foods [1]. Studying the effects of diet on health outcomes, including oral health, is more complicated. James Lind published a study in 1783 showing that vitamin C deficiency caused scurvy [2], but it took two more centuries for substantial progress to occur in the field of nutritional epidemiology. The recent explosion of studies relating nutrition, disease, and oral health is likely due to a combination of advances in designing and analyzing population health studies [3] including oral health measures [4], methods to measure diet [5, 6], and fast data processing. Recent studies leveraging advances in gene sequencing show that the human microbiome is much more diverse and complex than previously thought, and that its composition is correlated with the metabolic state, health, and disease [7]. Importantly, the human microbiome is affected by diet, and the oral microbiome and gut and human microbiome are connected [8–10]. These findings have opened a new window for future research.

**Figure 1:**
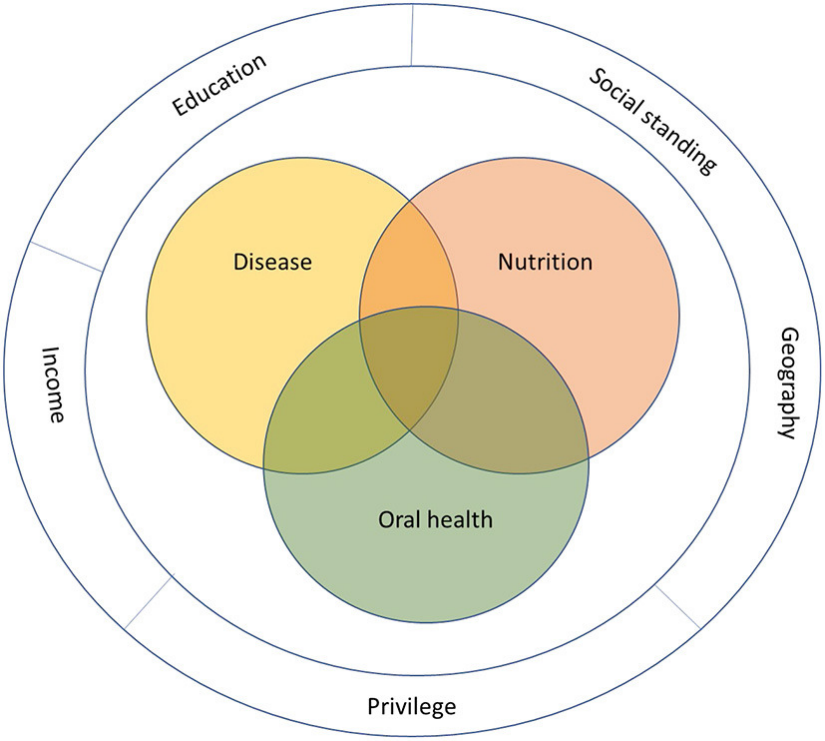
Context and interaction of nutrition, disease, and oral health.

At a population level, social and economic factors such as education, social standing, privilege, geography, and income affect both health outcomes and access to food, causing disparities in disease burden [11]. Globally, undernutrition continues to affect primarily mothers and children living in poor communities, while rates of obesity, diabetes, and chronic disease are rising [12–14]. On the other hand, the growing aging population has a unique set of circumstance—including multimorbidity, frailty, mobility, cognition, and loneliness—that potentially affect health and access to food [15]. This is the context within which nutrition researchers work (Figure 1).

The goal is to answer questions at the intersection of nutrition, oral health, disease, and their determinants that would inform clinical practice and policy. A substantial body of observational and mechanistic data exist relating various aspects of the relationships between diet, disease, and oral health. Poor oral health is associated with increased mortality [16, 17], heart disease [18], cancer [19], diabetes [20, 21], Alzheimer's disease [22], adverse birth outcomes [23], and other health conditions.

Diet is associated with oral and general health and affects the human microbiome. Changes in oral health are associated with changes in the oral and gut microbiome [8, 12], and dental treatment affects the oral microbiome [24]. Based on these data organizations including the American Diabetes Association [25], and American College of Obstetrics and Gynecology acknowledge that poor oral health is related to poor health outcomes [26], but neither of these organizations recommend dental treatment as a strategy to improve general health outcomes. The reason is that the effect of dental treatment in the prevention of general health outcomes is understudied.

Randomized controlled trials (RCT) are the gold standard to evaluate causality, but such studies are difficult to conduct in this area. For example, in an RCT designed to evaluate the effectiveness of dental treatment to prevent cardiovascular disease, dental treatment would need to be withheld from individuals in the control group for the time it would take for cardiovascular disease to develop. Restricting effective treatment for any length of time but particularly for the several years it takes for the outcome to develop would be ethically unacceptable [27]. Angrist showed that it was possible to leverage natural experiments using observational data to evaluate cause and effect [28]. The method is widely used in economics to evaluate the effect of policies, and Angrist and Imbens (a co-author on the seminal paper on the topic) received the 2021 Nobel Prize in economics for this contribution. McClellan et al. demonstrated the feasibility of using this approach in the medical field by comparing different treatments of myocardial infarction in relation to mortality using observational data [29]. More recently, Rigdon et al. used this method to assess the effect of the Supplemental Nutrition Assistance Program (SNAP) on body mass index (BMI) [30]. Robins proposed alternative approaches to evaluate the effect of treatment on health outcomes using observational data [31]. Hernan et al. reproduced the results of an RCT by applying these methods to observational data [32]. The Target Trial approach is exactly this—analyzing observational data to emulate a clinical trial [33, 34]. Given the difficulties in conducting RCTs in this field, we need to rely on good observational studies combined with these promising but underused alternative approaches understand the relationships between nutrition, disease, and oral health.

Racial disparities in relation to disease prevalence, oral health and diet are well-documented, but few studies have examined possible factors contributing to racial disparities in health outcomes. Kovesdy et al. found that Black individuals did not have a higher risk of death than White individuals in a large cohort of US veterans, but had a higher mortality risk in a similar sample representative of the general US population [35]. The US veterans had similar access to care irrespective of race, but Black persons have less access to care compared to their White peers in the general US population. Race was probably a proxy for access to care, which was unmeasured in the cohort of the general US population. Duggan and colleagues recently published a comprehensive update on this issue highlighting the limitations of using race in biomedical research and provided possible alternative approaches. There is growing recognition for the need to study health disparities in relation to their underlying determinants, such as access to care, discrimination, and social class for which race often serves as a crude proxy [36].

This is a particularly opportune time to study connections between nutrition, oral health, and disease because there are many unanswered questions. When we conducted a PubMed search with the terms “diet AND cardiovascular disease”, there were 74,590 results overall, of which 71% were from studies conducted from the year 2000 onwards (Table 1). A PubMed search with the terms “oral health AND cardiovascular disease” returned 15,625 results, 91% of which were after 2000 (Table 2). A PubMed search with the terms “diet AND cardiovascular disease AND oral health” returned 959 results overall, with a far larger percent (90%) after 2000 (Table 3). Studying the interrelatedness of nutrition, oral health, general health and their context is important because the results can be used in developing interventions and policies to improve oral and general health.

**Table 1:** Number of results in PubMed with search terms linking diet and health outcomes.

**PubMed search**	**All years**	2000 to 2022*
	***N***	***N*** **(%)**
Diet AND cardiovascular disease	74,590	53,029 (71)
Diet AND cancer	58,032	42,539 (73)
Diet AND diabetes	74,834	60,448 (81)
Diet AND hypertension	28,488	19,367 (68)
Diet AND pregnancy outcomes	5,182	4,521 (87)
Diet AND Alzheimer's disease	3,561	3,413 (96)
Diet AND obesity	90,685	77,388 (85)
Diet AND oral health	9,998	8,488 (85)
Diet AND periodontal disease	1,630	1,023 (63)

^*^Search conducted on June 29, 2022.

**Table 2:** Number of results in PubMed with terms linking oral and systemic health outcomes.

**PubMed search**	**All years**	2000 to 2022*
	***N***	***N*** **(%)**
Oral health AND cardiovascular disease	15,625	14,208 (91)
Oral health AND cancer	30,839	27,846 (90)
Oral health AND diabetes	16,120	14,910 (92)
Oral health AND hypertension	4,525	4,045 (89)
Oral health AND Alzheimer's disease	852	815 (96)
Oral health AND pregnancy outcomes	3,114	2,875 (92)
Oral health AND obesity	6,106	5,694 (93)

^*^Search conducted on June 29, 2022.

**Table 3:** Number of results in PubMed with terms linking diet, oral and systemic health outcomes.

**PubMed search**	**All years**	2000 to 2022*
Diet AND cardiovascular disease AND oral health	959	862 (90)
Diet AND cancer AND oral health	1,216	1,059 (87)
Diet AND diabetes AND oral health	2,421	2,145 (89)
Diet AND hypertension AND oral health	357	306 (86)
Diet AND pregnancy outcomes AND oral health	207	196 (95)
Diet AND Alzheimer's disease AND oral health	71	68 (96)
Diet AND obesity AND oral health	1,497	1,394 (93)

^*^Search conducted on June 29, 2022.

There are also a growing number of resources to address these questions. The complexity and cost of collecting primary data to answer these questions could have blocked this line of research, but increased availability of datasets for secondary analysis now make this possible. For example, Berkowitz et al. combined data from the 2011 National Health Interview Survey (NHIS) and Medical Expenditure Panel Survey (MEPS) to evaluate the relationship between SNAP program participation and health care costs [37]. The National Center for Health Statistics collects NHIS and MEPS data from a nationally representative sample and makes these data available for research. The National Health and Nutrition Examination Survey (NHANES) are publicly available data representative of the US population that several researchers have used [38]. Online resources such as a catalog of potential sources of datasets for secondary analyses are also available [39]. There have been substantial advances in methods to collect and analyze nutritional data, use observational data to emulate clinical trials, and evaluate mediators of causal effect.

Diet affects health including the health of teeth, gums, and tissues of the mouth, and is correlated with other lifestyle choices such as physical activity, and consumption of sugar sweetened beverages, alcohol, and betel nut chewing, which could be related to both oral and general health. The Oral Health and Nutrition section of Frontiers in Oral Health will consider studies evaluating:

- the impact of oral health and tooth loss on diet
- the impact of nutrition on health including bone, teeth and soft tissues in the mouth and on overall health
- common nutritional risk and preventive factors of oral disease and health
- taste and nutrition
- socioeconomic disparities and food insecurity in relation to oral and systemic health
- promotion of healthy diet and referral to dietitians
- lifestyle and its impact on oral and general health.

Studying these connections can help to translate research findings into clinical practice and policy to improve oral and overall health.

## Author contributions

The author confirms being the sole contributor of this work and has approved it for publication.

## Conflict of interest

The author declares that the research was conducted in the absence of any commercial or financial relationships that could be construed as a potential conflict of interest.

## Publisher's note

All claims expressed in this article are solely those of the authors and do not necessarily represent those of their affiliated organizations, or those of the publisher, the editors and the reviewers. Any product that may be evaluated in this article, or claim that may be made by its manufacturer, is not guaranteed or endorsed by the publisher.
